# Inflammatory breast cancer defined: proposed common diagnostic criteria to guide treatment and research

**DOI:** 10.1007/s10549-021-06434-x

**Published:** 2022-01-01

**Authors:** R. Jagsi, G. Mason, B. A. Overmoyer, W. A. Woodward, S. Badve, R. J. Schneider, J. E. Lang, M. Alpaugh, K. P. Williams, D. Vaught, A. Smith, K. Smith, K. D. Miller, Susan G. Komen-IBCRF IBC Collaborative in partnership with the Milburn Foundation

**Affiliations:** 1 University of Michigan, Ann Arbor, MI USA; 2 Inflammatory Breast Cancer Research Foundation, West Lafayette, IN USA; 3 Susan G. Komen Advocates in Science, Dallas, TX USA; 4 Dana Farber Cancer Institute, Boston, MA USA; 5 University of Texas MD Anderson Cancer Center, Houston, TX USA; 6 Indiana University Melvin and Bren Simon Comprehensive Cancer Center, 535 Barnhill Drive, RT 473, Indianapolis, IN 46202 USA; 7 New York University School of Medicine, New York, NY USA; 8 Cleveland Clinic, Cleveland, OH USA; 9 Rowan University, Glassboro, NJ USA; 10 North Carolina Central University, Durham, NC USA; 11 Susan G. Komen, Dallas, TX USA

**Keywords:** Inflammatory breast cancer, Clinical diagnosis, Pathology, Molecular markers, Diagnostic criteria

## Abstract

**Purpose:**

Inflammatory breast cancer is a deadly and aggressive type of breast cancer. A key challenge relates to the need for a more detailed, formal, objective definition of IBC, the lack of which compromises clinical care, hampers the conduct of clinical trials, and hinders the search for IBC-specific biomarkers and treatments because of the heterogeneity of patients considered to have IBC.

**Methods:**

Susan G. Komen, the Inflammatory Breast Cancer Research Foundation, and the Milburn Foundation convened patient advocates, clinicians, and researchers to review the state of IBC and to propose initiatives to advance the field. After literature review of the defining clinical, pathologic, and imaging characteristics of IBC, the experts developed a novel quantitative scoring system for diagnosis.

**Results:**

The experts identified through consensus several “defining characteristics” of IBC, including factors related to timing of onset and specific symptoms. These reflect common pathophysiologic changes, sometimes detectable on biopsy in the form of dermal lymphovascular tumor emboli and often reflected in imaging findings. Based on the importance and extent of these characteristics, the experts developed a scoring scale that yields a continuous score from 0 to 48 and proposed cut-points for categorization that can be tested in subsequent validation studies.

**Conclusion:**

To move beyond subjective ‘clinical diagnosis’ of IBC, we propose a quantitative scoring system to define IBC, based on clinical, pathologic, and imaging features. This system is intended to predict outcome and biology, guide treatment decisions and inclusion in clinical trials, and increase diagnostic accuracy to aid basic research; future validation studies are necessary to evaluate its performance.

**Supplementary Information:**

The online version contains supplementary material available at 10.1007/s10549-021-06434-x.

## Introduction

Little progress in improving inflammatory breast cancer (IBC) patient outcomes has been made since Lee and Tannebaum [1] first described *inflammatory carcinoma of the breast* in 1924. While awareness of breast cancer in general has increased dramatically, awareness of inflammatory breast cancer continues to lag. Public health campaigns encourage women to seek evaluation of breast masses or nipple discharge, but often fail to mention changes in skin color or texture. Professional articles often target the oncology community, rather than primary care community, neglecting the practitioners most likely to be the patient’s first contact. Reviews commonly stress the rarity of inflammatory breast cancer, leading many to dismiss it as a possible diagnosis. Delays in diagnosis and referral for appropriate treatment are all too common (see text box).

Similarly, scientific characterization remains rudimentary. Inflammatory breast cancer remains a clinical diagnosis with no pathognomonic hallmark. With the variability in presentation among individuals, even experienced clinicians may disagree on the diagnosis. Physicians participating in tumor boards frequently question, “Is the skin thickening merely a local manifestation of extensive nodal involvement? Is the erythema diffuse or focal? Did it precede biopsy? Did inflammatory changes develop secondarily in an otherwise neglected primary?” In short, physicians argue whether the breast is *inflamed enough* to be considered inflammatory breast cancer. Yet, despite the moniker, inflammatory breast cancer seems to lack widespread expression of biomarkers of inflammation.

To tackle these challenges, Susan G. Komen in partnership with the Inflammatory Breast Cancer Research Foundation and the Milburn Foundation assembled a panel of experts to focus on inflammatory breast cancer. The initial charge of the Susan G. Komen IBC Focus Group was broad – review the state of IBC care and research globally and propose specific initiatives to move the field forward. Discussions during our first meeting highlighted the limitations of the formal definition of IBC. Without an unambiguous definition, both patient care and research suffer, diagnosis remains subjective, and treatments vary. IBC patients are commonly allowed to participate in more general breast cancer trials, but their small numbers preclude meaningful subset analyses. Trials specifically focused on IBC are scant—both due to the rarity of the disease and the difficulty of defining it. Further, those IBC-specific clinical trials may be unintentionally underpowered by the inclusion of subjects with locally advanced, non-inflammatory breast cancer because of mistakes or simple differences in subjective categorization that lead them to be misclassified as having IBC. Together, these challenges limit the ability to elucidate the true efficacy of therapy for IBC and motivate the development of the quantitative definition as proposed here. Similarly, the search for a molecular *sine qua non* or unifying pathway aberration underlying IBC is hindered by inclusion of biospecimens from subjects with a different disease (namely non-IBC breast cancer) because of imprecision in the subjective definitions of what constitutes IBC. Our goal was to move beyond the ambiguous “clinical diagnosis” to more objective diagnostic criteria. Here we propose a more nuanced definition of IBC, based on a review of clinical, pathologic, and molecular features.

The 8th edition of the American Joint Committee on Cancer (AJCC) staging manual continues to define IBC based upon the presence of diffuse erythema and edema involving a third or more of the breast after confirmation of an invasive breast cancer. The diagnosis of IBC is categorized as clinical stage T4d [2]. With increased awareness of IBC, some patients come to medical attention at earlier times in the course of the disease, when clinical characteristics may not be as profound as in prior eras [3]. In addition, the recent establishment of high-volume IBC clinical centers has broadened our understanding of the differences in presentation. Therefore, we propose and describe a diagnostic scoring system developed by expert consensus that accounts for the variable presentation of IBC (Table 1) and warrants validation in future studies.

**Table 1:** Proposed scoring system for inflammatory breast cancer diagnosis

Characteristic	Score			Priority factor (multiplier)
	3	2	1	
Timing of signs/symptoms	≤3 months	3–6 months	>6 months	×3
Skin changes	Any peau d’orange	Skin edema/thickening* ver ≥ 1/3 of the breast	Focal skin edema/thickening^*^ (<1/3 of the breast)	×3
Swelling/engorgement of the breast	Clinically apparent enlargement of the breast or new asymmetry in breast size	*Intentionally blank; patients receive either a score of 3 or 1 for this characteristic*	Breast edema identified on imaging but not clinically detectable	×3
Erythema or other skin discoloration: pink, red, darkened, bruising/purplish, or serpiginous in character	Complete or near complete involvement of the breast	Not nearly complete but greater than minimal involvement of the breast	Minimal involvement or ambiguous color change	×2
Nipple abnormalities	New nipple inversion	New nipple flattening or other asymmetry	Crusting of the nipple/areola without other nipple changes	×2
Lymphatic tumor cell emboli	Dermal lymphatic emboli present (without evidence of direct involvement of the dermis or epidermis)	Non-dermal lymphatic emboli present (breast parenchyma or stroma)	*Intentionally blank; patients receive either a score of 3 or 2 for this characteristic*	×2
Breast imaging	Diffuse involvement of breast parenchyma (with or without dominant mass)	*Intentionally blank; patients receive either a score of 3 or 1 for this characteristic*	Enlargement of non-axillary nodes (internal mammary, supraclavicular, subpectoral, etc.)	×1

Clinical, pathologic, and imaging characteristics are listed in rows with a graded scoring system of 1–3 listed in columns, where 3 is definitively associated with inflammatory breast cancer and 1 less specific for IBC compared with non-inflammatory locally advanced breast cancer. If a characteristic it is totally absent, enter a score of zero. If statements in multiple columns describe the patient presentation, the highest scoring column should be selected. The priority factor in the far-right column represents how some characteristics are more heavily weighted for inflammatory breast cancer and thus represent a multiplying factor. The score for each characteristic is multiplied by the priority factor, then the subtotals of all the characteristics are added together to yield a total score (Total IBC Value). The Total IBC Value provides a score for use in identifying inflammatory breast cancer. Boxes marked “Intentionally blank” are not factored into the score. Proposed classifications of the Total IBC Scores are DEFINITELY IBC (total score ≥ 42); STRONG POSSIBILITY of IBC (total score 25–41); WEAK POSSIBILITY of IBC (total score 14–24); and NOT IBC (total score < 14)

*Skin thickening may be assessed on clinical examination or observed on breast imaging

## Clinical presentation

### Timing of the onset of symptoms/signs of IBC

The hallmark of IBC is the sudden onset of signs and symptoms within a 6-month period prior to the diagnosis of invasive disease [4, 5]. This rapid onset of clinical features distinguishes IBC from non-inflammatory locally advanced breast cancer (LABC) arising from a more indolent subtype of invasive breast cancer, often neglected for years [6]. It should be noted that signs and symptoms of IBC which occur within a 6-month time frame, but do not result in a pathologic diagnosis of cancer until after 6 months, should still support the diagnosis of IBC. The key is timing of the *onset* of the symptoms detailed below. Delay in diagnosis is not uncommon, and such delays alone should not alter the diagnosis.

### Thickening or edema of the skin of the breast

As with erythema, thickening of the skin of the breast is also understood to be a consequence of dermal lymphatic involvement with tumor emboli. The edema or thickening can also vary in extent and severity, with the characteristic associated with the most gravity being *peau d’orange*, having the texture and/or appearance of an orange peel [7, 8]. There can be ridges or indurations palpable within the skin, corresponding to the areas of lymphatic involvement, which are not contiguous. Ulceration or nodularity of the skin is less likely associated with IBC than with the more indolent LABC [9]. Whereas evidence of skin thickening is best seen with Magnetic Resonance (MR) imaging, conventional mammography and ultrasound can also detect this feature [10–12].

### Edema or swelling of the breast

Asymmetrical enlargement of the affected breast has been used as a criterion for the diagnosis of IBC since 1978 [3]. A palpable mass within the breast is frequently absent, yet the extent of tumor involvement and associated edema within the breast is often demonstrated by breast imaging. The clinical history may support the presence of breast edema by describing an ill-fitting garment that causes pain and which may leave indentations on the breast.

### Erythema of the skin of the breast

The erythema associated with IBC is believed to be due to dilated capillaries associated with tumor emboli within the papillary or reticular dermis of the skin of the breast. The hyperemia is functional and can wax and wane over time and occasionally disappear on examination [13]. This feature of IBC can be described in several ways, including bruising or ecchymosis, various gradations of redness (faintly pink to bright red), confluent, or serpiginous [14, 15]. In some women the skin may be obviously changed compared to baseline but may not appear red at all. The extent of skin involvement may also vary over time. IBC has a higher incidence among individuals with highly pigmented skin, e.g., those of African, South Asian, and Arabic descent, so it is important to broaden the interpretation of “skin erythema” beyond simply “redness” [16].

### Nipple abnormalities

**Fig. 1:**
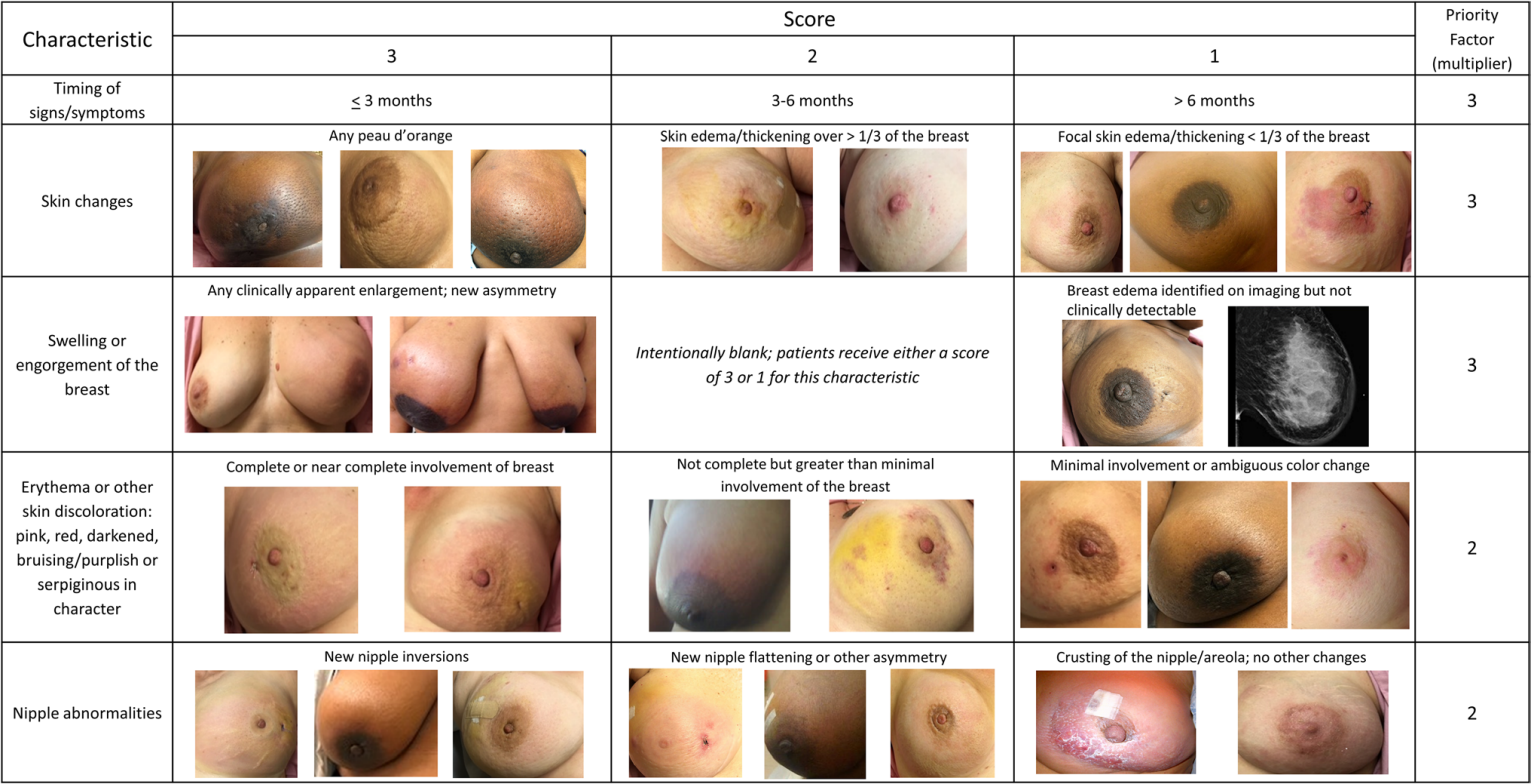
Clinical characteristics of Inflammatory Breast Cancer. Clinical characteristics and variations of established conditions to support the diagnosis of Inflammatory Breast Cancer as measured by the proposed scoring system

Changes in the nipple may be a characteristic of IBC, including nipple inversion, nipple flattening, or crusting of the nipple/areolar complex [17]. However, as many other benign or malignant etiologies may explain nipple abnormalities, additional diagnostic criteria are required for a diagnosis of IBC. Examples of these clinical criteria are shown in Fig. 1.

## Pathophysiology

### Lymphovascular tumor cell emboli

Dermal lymphovascular tumor cell emboli are regarded as the classic histological feature of IBC [18]. Secondary to lymphovascular emboli are skin changes (e.g., erythema, edema, *peau d’ orange*) caused by occluded vessels, rendering IBC a clinicopathological diagnosis [19]. While dermal lymphatic emboli are common, involvement is often patchy and can be missed with a single punch biopsy. Although considered classic, pathologic confirmation of dermal lymphatic emboli is not required. In a minority of IBC cases, skin changes are present with patent lymphatics and no visible dermal lymphatic emboli. Although not considered a classic feature, lymphatic emboli in the breast parenchyma or stroma may be present and is also indicative of IBC. Similarly, lack of pathologically identified stromal or parenchymal lymphovascular emboli do not exclude a diagnosis of IBC.

Tumor emboli represent focal events and whether in the dermis or breast parenchyma, are patchy and can be missed when pathologic evaluation is limited. If no emboli are seen in a single sample and the diagnosis remains in question, either an additional biopsy or analysis of multiple levels should be considered. Tumor cell emboli often appear smaller than the vessel due to retraction from the lymphatic channels [20]. The vessels typically do not exhibit any thickening or secondary changes, although sometimes one can note lymphocytic cuffing around these vessels.

**Fig. 2:**
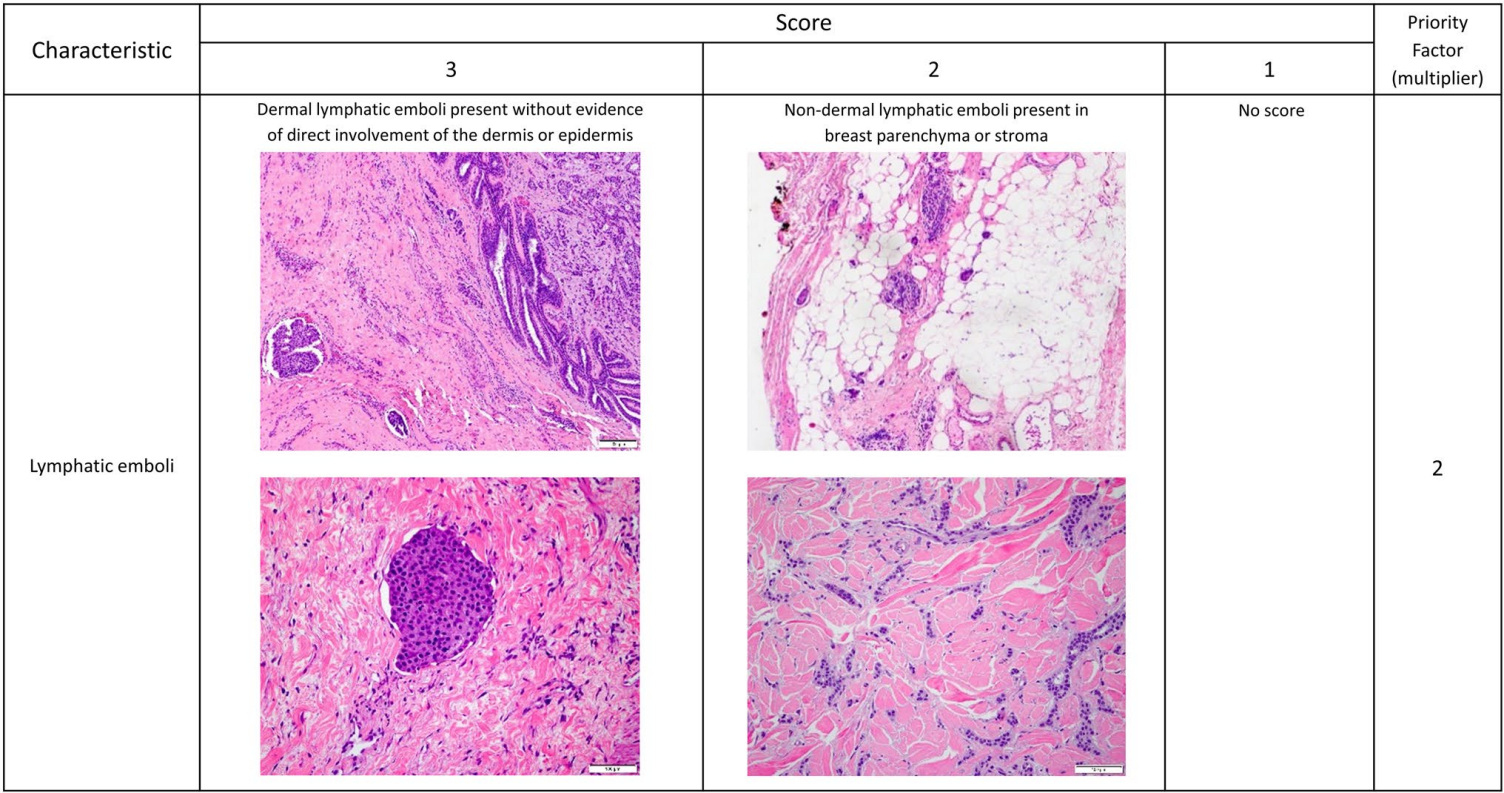
Pathologic characteristics for Inflammatory Breast Cancer. Variations of the pathologic presentation of lymphatic emboli, represented in H&E tissue staining, to support the diagnosis of Inflammatory Breast Cancer as measured by the proposed scoring system

In contrast to dermal lymphatic involvement, direct skin involvement and invasion of the dermis is extremely unusual in IBC. Direct skin involvement is common in non-inflammatory LABC, particularly in patients with longstanding neglected disease [21]. Similarly, ulceration of the epidermis and/or dermal scarring is not characteristics of IBC and, if present, should raise questions about an IBC diagnosis. Examples of these pathologic characteristics are shown in Fig. 2.

We considered multiple additional pathologic and molecular characteristics (Supplemental Table 1) as well as genomic classifiers. While the panel expressed enthusiasm for continued study of several potential molecular markers, in general samples sizes were small and validation lacking or inconsistent. Consequently, none were included in the diagnostic scoring system we proposed and thus are not discussed further here. Interested readers are directed to one of several recent reviews of IBC [22–24].

## Breast imaging characteristics

**Fig. 3:**
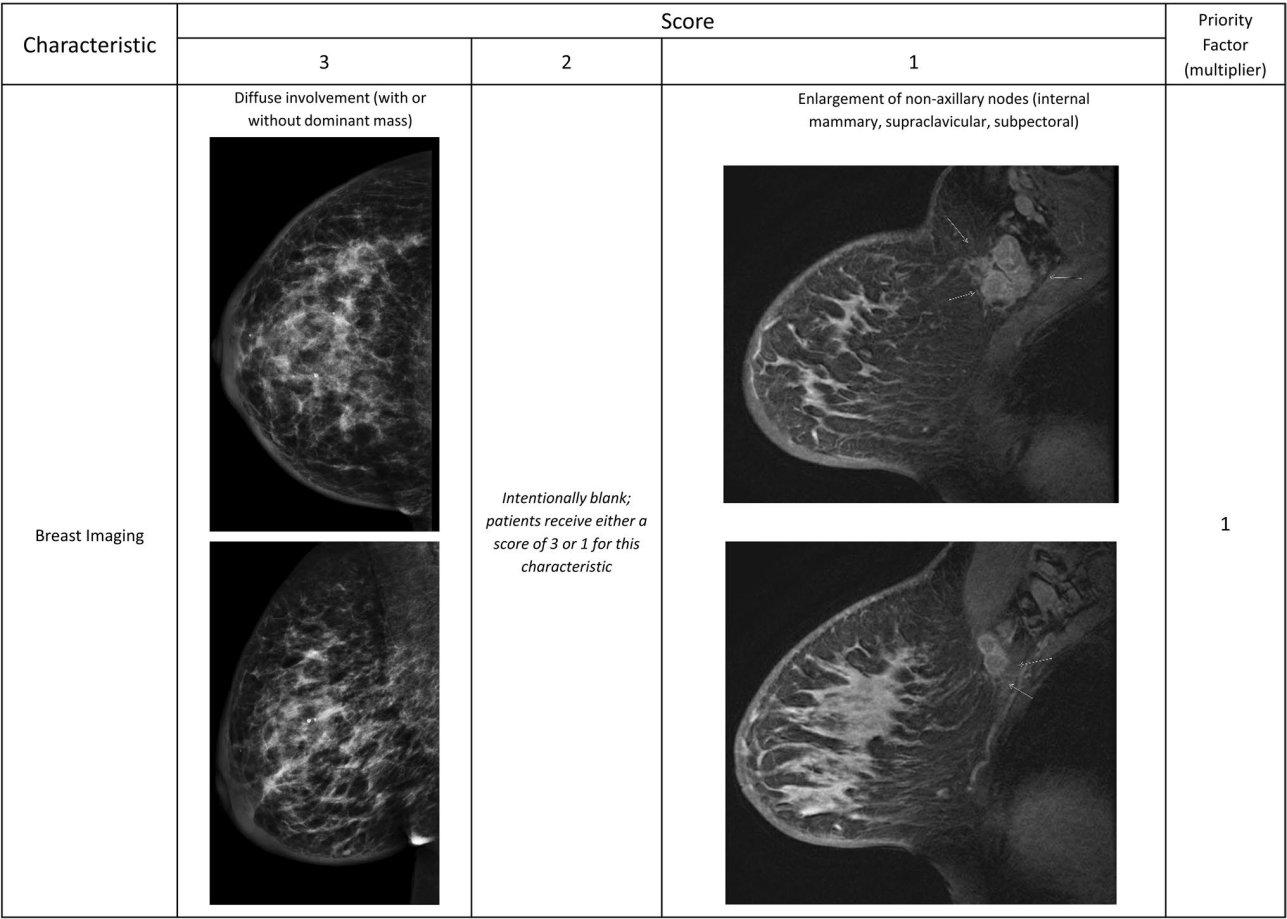
Imaging characteristics of Inflammatory Breast Cancer. Variations of the breast imaging to support the diagnosis of Inflammatory Breast Cancer as measured by the proposed scoring system

Mammographically, IBC often presents as diffuse increased density within the breast, whereas MR imaging describes these changes as extensive non-mass-like enhancement [25]. In general, in cases of IBC, breast imaging should demonstrate more diffuse involvement, exhibiting evidence of disease beyond a focal mass. Breast imaging will often demonstrate extensive regional nodal involvement outside of the ipsilateral axilla, such as the subpectoral, supraclavicular, and internal mammary regions. MR is preferred whenever IBC is suspected but is not required for diagnosis. Examples of these imaging criteria are shown in Fig. 3.

## Assessment and diagnosis

We propose a composite definition of IBC based upon a graded scoring system of 1–3, where 3 is definitively associated with IBC and 1 less specific for IBC compared with non-inflammatory LABC. Some characteristics were considered more important and thus each characteristic was assigned a priority factor determined based on expert consensus of the co-authors. To determine the total score or Total IBC Value, the score of each characteristic is multiplied by the priority factor, and the subtotals are added together to obtain the Total IBC Value. If a characteristic is completely absent, a score of zero should be entered. Therefore, the score can range from 0 to 48. As initial cut-points for categorizing scores, based on the expert consensus of the co-authors, we propose the following classifications of the Total IBC Values:

- DEFINITELY IBC (total score ≥ 42)
- STRONG POSSIBILITY of IBC (total score 25–41)
- WEAK POSSIBILITY of IBC (total score 14–24)
- NOT IBC (total score < 14)

### Clinical use

The purpose of applying the proposed IBC Scoring System (Table 1) to clinical practice is to facilitate a diagnosis of IBC in the most prompt, accurate, and consistent manner possible. The application of this diagnostic tool can be used at any point in the disease presentation, e.g., before or after a trial of antibiotics, and without regard to the timing of a breast biopsy. Because of the variable nature of this disease, the clinical breast exam may change over time. This may impact the total score; therefore, we recommend that the diagnosis be based on the highest score observed, regardless of the persistence of the characteristic. A higher score is associated with a more classic and obvious diagnosis of IBC.

A complete evaluation of any potential clinical presentation of IBC includes a skin punch biopsy and breast imaging, MR imaging being the most definitive modality [10, 25]. A skin punch biopsy of the area of greatest change, i.e., erythema or edema (peau d’orange) should always be attempted, but like the AJCC diagnostic criteria for IBC, the presence of dermal lymphatic emboli is not required for the diagnosis using the proposed IBC Scoring System, provided other criteria can be assessed and a score of > 42 has been documented. Skin punch biopsy is strongly recommended for all patients with a score < 42 who have not had a previous skin biopsy. If skin ulceration is present, a biopsy of this area is discouraged since it is unlikely to be diagnostic [16].

An example of the application of the proposed IBC Scoring System is outlined in Table 2. We recommend that patients with a ‘strong possibility of IBC’ be treated with trimodality therapy (preoperative systemic therapy based on disease phenotype, mastectomy, and radiation therapy) identical to patients with definite IBC [26]. The choice of treatment of those patients with a lesser possibility of IBC will be driven by disease phenotype, anatomic extent, and patient goals. Trimodality therapy may be appropriate for many patients in this diagnostic category but cannot be mandated.

**Table 2:** Example application of the proposed IBC scoring system

Characteristic	Score	X priority factor	= IBC value
Timing of signs 3–6 months	2	3	6
Skin change to peau d`orange	3	3	9
Swelling identified by imaging	1	3	3
Skin discoloration partial involvement of breast	2	2	4
Nipple flattening or other asymmetry	2	2	4
Dermal lymphatic emboli present in pathology samples	3	2	6
Breast imaging shows diffuse involvement of breast	3	1	3
Total score			35

Using the proposed diagnostic criteria and scoring system, a value was determined for a hypothetical patient based on the clinical, pathologic, and imaging characteristics observed, their score values, and the priority factor of each characteristic. The Total IBC Value is represented at the bottom right; a score of 35 would represent strong possibility of IBC

## Conclusion

We acknowledge that our proposed IBC Scoring System requires validation to evaluate its performance, refinement of the criteria, their weight in the total score, or the thresholds for diagnosis may require refinement. If accurate, we expect patients with IBC based on these criteria to have a different clinical course and outcome compared to patients with LABC that fail to meet our definition. We ourselves have begun retrospective validation using large clinical trial datasets, although missing data may make this challenging. Concurrently we will work with large academic and community centers to prospectively validate the scoring system, making modifications as new data emerge. Ultimately, we envision using the final disease classification and scoring system to develop a staging system specific to IBC.

While the scoring system with priority factors is quantitative, most of the clinical criteria remain subjective. Ultimately, discovery and validation of distinct molecular and/or genetic factors or pathways that distinguish IBC from non-inflammatory LABC will be needed to truly eliminate subjectivity from the diagnosis. Here, we also see the utility of our diagnostic criteria. Discovery studies should initially focus on those with definite IBC, the group with the most homogenous and extreme clinical phenotype. Once identified, promising markers should be explicitly studied in patients with lower scores to better determine their sensitivity and specificity. Finally, preclinical models for IBC should be developed and evaluated based on their fidelity to the phenotype captured in our diagnostic criteria.

## Supplementary Information

Below is the link to the electronic supplementary material.

Supplementary file1 (DOCX 18 kb)

## Data Availability

No datasets were generated or analyzed in the creation of this review manuscript.
