# Supplementary material for: Inflammatory breast cancer defined: proposed common diagnostic criteria to guide treatment and research
Source: Breast Cancer Res Treat. 2022 Jan 1;192(2):235–43. doi: 10.1007/s10549-021-06434-x (PMC8926970; doi:10.1007/s10549-021-06434-x)
Supplement: Supplementary file 1 — Supplementary file1 (DOCX 18 kb) [file 10549_2021_6434_MOESM1_ESM.docx]

**Supplementary Table 1:** Potential Molecular Markers of Inflammatory Breast Cancer

| **Biomarker** | **Characterization – Magnitude/Direction of Change** |
| --- | --- |
| E-cadherin ^1-3^ | Overexpression |
| p-Stat3 ^4,5^ | High levels of phosphorylated Stat3 in IBC samples |
| JAK2 ^4,5^ | Activated |
| VEGF-C and -D ^6^ | Over-expression |
| LYVE-1 ^6^ | Over-expression |
| Macrophage Marker ^7,8^ | Increased |
| RhoC ^9,10^ | Over-expressed |
| WISP3 ^9,10^ | Loss of expression |
| Calveolin proteins 1 and 2 ^11^ | Over-expressed |

A panel of biomarkers that are strongly associated with IBC in preliminary reports. Validation in independent data sets is either lacking or the association with IBC has been inconsistent. While current data does not support incorporation of these factors into the diagnostic criteria, they warrant further study in cohorts defined by the proposed diagnostic criteria.

**References**

1. Kleer CG, van Golen KL, Braun T, and Merajver SD (2001) Persistent E-cadherin expression in inflammatory breast cancer. Mod

Pathol 14:458-64. <https://doi.org/10.1038/modpathol.3880334>

2. Colpaert GC, Vermeulen PB, Benoy I, Soubry A, Van Roy F, van Beest P, Goovaerts G, Dirix LY, Van Dam P, Fox SB, Harris AL and Van Marck EA (2003) Inflammatory breast cancer shows angiogenesis with high endothelial proliferation rate and strong E-cadherin expression. Br J Cancer 88, 718–725. <https://doi.org/10.1038/sj.bjc.6600807>

3. Tomlinson JS, Alpaugh ML and Barsky SH (2001) An Intact Overexpressed E-cadherin/α,β-Catenin Axis Characterizes the Lymphovascular Emboli of Inflammatory Breast Carcinoma. Cancer Res 61;13, 5231-5241. CNREA8; ISSN: 0008-5472

4. Overmoyer BA, Almendro V, Shu S, Peluffo G, Park SY, Nakhlis F, JBellon JR, EYeh ED, Jacene HA, Hirshfield-Bartek J and Polyak K (2012) JAK2/STAT3 activity in inflammatory breast cancer supports the investigation of JAK2 therapeutic targeting. Cancer Res. 72 (24 Suppl): Abstract nr P4-06-01. https://doi.org/10.1158/0008-5472.SABCS12-P4-06-01

5. Jhaveri K, Teplinsky E, Silvera D, Valeta-Magara A, Arju R, Gishuddin S, Sarfraz Y, Alexander M, Darvishian F, Levine PH, Hashmi S, Zolfaghari L, Hoffman HJ, Singh B, Goldberg JD, Hochman T, Formenti S, Esteva FJ, Moran MS and Schneider RJ (2016) Hyperactivated mTOR and JAK2/STAT3 Pathways: Molecular Drivers and Potential Therapeutic Targets of Inflammatory and Invasive Ductal Breast Cancers After Neoadjuvant Chemotherapy. Clin Breast Cancer 16:113-22 e1, 2016. https://doi.org/10.1016/j.clbc.2015.11.006

6. Van der Auwera I, Van Laere SJ, Van den Eynden GG, Benoy I, van Dam P, Colpaert CG, Fox SB, Turley H, Harris AL, Van Marck EA, Vermeulen PB and Dirix LY (2004) Increased angiogenesis and lymphangiogenesis in inflammatory versus noninflammatory breast cancer by real-time reverse transcriptase-PCR gene expression quantification. Clin Cancer Res 10:7965-71. https://doi.org/10.1158/1078-0432.CCR-04-0063

7. Reddy JP, Atkinson RL, Larson R, Burks JK, Smith D, Debeb BS, Ruffell B, Creighton CJ, Bambhroliya A, Reuben JM, Van Laere SJ, Krishnamurthy S, Symmans WF, Brewster AM and Woodward WA (2018) Mammary stem cell and macrophage markers are enriched in normal tissue adjacent to inflammatory breast cancer. Breast Cancer Res Treat 171:283-293. https://doi.org/10.1007/s10549-018-4835-6

8. Valeta-Magara A, Gadi A, Volta V, Walters B, Arju R, Giashuddin S, Zhong H and Schneider RJ (2019) Inflammatory Breast Cancer Promotes Development of M2 Tumor-Associated Macrophages and Cancer Mesenchymal Cells through a Complex Chemokine Network Cancer Res 79:3360–71. https://doi.org/10.1158/0008-5472.CAN-17-2158

9. van Golen KL, Davies S, Wu ZF, Fang Wang Y, Bucana CD, Root H, Chandrasekharappa S, Strawderman M, Ethier SP and Merajver SD (1999) A novel putative low-affinity insulin-like growth factor-binding protein,

LIBC (lost in inflammatory breast cancer), and RhoC GTPase correlate with the inflammatory breast cancer phenotype. Clin Cancer Res 5:2511-9. Print ISSN 1078-0432

10. Celina G Kleer, Yanhong Zhang, Quintin Pan, Gary Gallagher, Mei Wu, Zhi-Fen Wu & Sofia D Merajver. WISP3 and RhoC guanosine triphosphatase cooperate in the development of inflammatory breast cancer. Breast Cancer Res 6, R110 (2004). https://doi.org/10.1186/bcr755

11. Van den Eynden GG, Van Laere SJ, Van der Auwera I, Merajver SD, Van Marck EA, van Dam P, Vermeulen PB, Dirix LY and van Golen KL (2006) Overexpression of caveolin-1 and -2 in cell lines and

in human samples of inflammatory breast cancer. Breast Cancer Res Treat 95:219-28. https://doi.org/10.1007/s10549-005-9002-1
